# Determinants of costs of human papillomavirus vaccine delivery in six low- and middle-income countries

**DOI:** 10.1016/j.jvacx.2024.100534

**Published:** 2024-07-27

**Authors:** Mercy Mvundura, Rose Slavkovsky, Frédéric Debellut, Teddy Naddumba, Clint Pecenka, D. Scott Lamontagne, HPV vaccine cost of delivery study collaborators

**Affiliations:** a PATH, Seattle, USA; b PATH, Geneva, Switzerland; c PATH, Kampala, Uganda; d JSI Research & Training Institute, Inc., Arlington, USA. Formerly with PATH, Seattle, USA

**Keywords:** Cost determinants, Cost drivers, Human papillomavirus vaccine, Delivery costing, Vaccine economics, Immunization costing, Operational research, Low- and middleincome countries

## Abstract

Evidence on determinants of vaccine delivery costs can inform program design and planning. Given the dearth of this evidence for human papillomavirus (HPV) vaccine, we conducted an analysis to identify programmatic and operational factors that are statistically associated with variations in economic costs for HPV vaccine delivery, within and across six low- and middle-income countries.

HPV vaccine program operations and cost data were collected from Ethiopia, Guyana, Rwanda, Senegal, Sri Lanka, and Uganda. An ordinary least square regression analysis was done using data from 279 health facilities in these six countries. We ran country-specific and pooled multivariate linear regressions. A conditional regression including 228 facilities was also run. The dependent variable was the estimated total economic costs for HPV vaccine delivery per facility, excluding vaccine procurement costs. Explanatory variables included number of HPV vaccine doses delivered; numbers of vaccination sessions conducted, and schools served; distance traveled by health workers for vaccine delivery; intensity of conducting program activities; human resource (health workers, school staff, etc.) utilization rates; and categorical variables indicating whether per diems were paid, and for country-specific dummies;

Explanatory variables such as the number of program activities or meetings held, receipt of per diems, and utilization rates of health workers, were all positively and statistically significantly associated with economic costs in the pooled sample, for both the unconditional and conditional regressions. Variables such as the doses delivered, and number of sessions conducted were statistically significant in the unconditional regression. The within-country regression found that only variations in utilization rates of health workers were statistically significant in all countries.

Our analysis provides evidence to HPV vaccination program stakeholders on which program context variables impact costs, which can inform program adjustment to improve cost efficiency, especially as programs managers work to revitalize and rebuild HPV vaccine coverage after the COVID-19 pandemic.

## Introduction

Recent studies have shown that the costs to deliver human papillomavirus (HPV) vaccines vary within and across low- and middle-income countries (LMIC) [1], [2], [3], but there is limited evidence on the factors that explain these cost differences. A handful of previous studies have explored the determinants of economic cost for routine infant vaccination programs [4], [5], [6], [7], [8] but there is a dearth of evidence for HPV vaccination programs targeted to adolescents [1]. Although the evidence from routine infant vaccine delivery is useful, it may fail to capture the drivers of HPV vaccine delivery required for effective and budget-conscious program planning. For example, most routine HPV vaccination services in LMICs are provided in school-based or outreach settings (in which health workers must travel to vaccination sites) and so may have different cost determinants than infant vaccines, which are predominantly provided in facility-based settings. As such, factors such as the number of schools served, and distance traveled by health workers to deliver HPV vaccinations may impact program costs.

There are other unique features of routinized HPV vaccination programs, which deliver vaccines to adolescents year-on-year. Payment of per diems to vaccination teams for service delivery at schools or outreach is common for HPV vaccination programs, which is not typically done for routine immunization service delivery at health facilities, though per diems may be paid for outreaches for routine immunization. Timing of HPV vaccination delivery also differs by country, with some countries having year-round delivery and others concentrating vaccination sessions at two time points, consistent with the two-dose schedule used in many countries [9]. Programs with year-round HPV vaccine delivery may have a larger number of HPV vaccination sessions held by each health facility, compared to those concentrating service delivery to fixed time points, impacting costs.

Due to the COVID-19 pandemic, global HPV vaccine coverage dropped by 15 %; this was the largest decline for any vaccine during the pandemic [10]. As HPV vaccination programs work to revitalize and expand coverage, evidence on cost drivers can inform program planning and decision-making by helping to identify the largest cost drivers and areas for greater cost efficiency without compromising program quality.

We recently completed a study in six low- and middle-income countries that have routine HPV vaccination programs that are past the HPV vaccine introduction years. The completed study evaluated the operational context and costs of HPV vaccine delivery in these countries [11]. Building on that study, this research aimed to identify determinants of HPV vaccine delivery costs at the health facility level within and across countries using data from these six national programs.

## Methods

### Data used for the analysis

We conducted secondary analysis of data from a primary mixed-methods study that evaluated the operational context and economic and financial costs of HPV vaccine delivery in six countries: Ethiopia, Guyana, Rwanda, Senegal, Sri Lanka, and Uganda [11]. Operational research and microcosting data used in this analysis was collected and analyzed using standardized and consistent methods across the study countries. HPV vaccination program activities such as program planning and management, social mobilization, training, vaccine collection and storage, service delivery, crisis management, and waste disposal were evaluated. We calculated economic costs for each HPV vaccination program activity conducted by the health facilities; these economic costs included financial and opportunity costs [12]. Financial costs account for expenditures with direct financial outlays such as per diems paid, venue rentals and meals for meetings, expenditures for travel such as vehicle rental and costs for public transport, expenditures for fuel for vehicles and other equipment and vehicle maintenance, and distribution of materials for social mobilization. Opportunity costs account for costs of using existing resources and include time costs for health workers/vaccinators, support staff, and non-health workers, as well as the annualized cost for vehicles and equipment, such as refrigerators for vaccine storage and incinerators for waste disposal. Total annual economic costs per health facility were estimated in 2019 US$, excluding the value of vaccines and immunization supplies such as syringes, and from the health system perspective. The operational research was embedded in the costing study and described the HPV vaccination program implementation, assessing what was done, how, how often, and by whom. These were tabulated as frequencies and counts, depending on the variable evaluated. The reference period for the evaluation was 2019, targeting a period before the COVID-19 pandemic to avoid capturing the impact of the pandemic on HPV vaccine delivery context and costs. We used random sampling to select the health facilities. The sample sizes by country were 60 for Ethiopia, 43 for Guyana, 42 for Rwanda, 56 for Senegal, 30 for Sri Lanka, and 66 for Uganda, for a total of 297 health facilities.

For this secondary analysis, we focused on costs at health facilities for several reasons. Activities done at the health facility level contributed the largest share to the aggregated economic costs per dose, accounting for between 54 % and 95 % of the costs for HPV vaccine delivery [11]. Secondly, the determinants of costs at the health facility level where service delivery occurs are likely not the same as program activities conducted by administrative health offices (e.g., district, regional or national level) who do not provide HPV vaccination services. Lastly, small sample sizes for administrative health offices preclude an in-depth analysis of cost drivers at this level.

### *Regression* function

For this analysis, we applied the following regression function based on economic theory in which costs are a function of quantity and other factors, as described in previous analyses evaluating the determinants of costs [5], [6],

$${\log C}_{i}=\;\beta_{0\;}+\;\beta_{1\;\;}\times log\;\left(Q_{i\;}\right)\;+\;\beta_{n\;}\times Z_{n\;}+\;\varepsilon$$

where C is the cost at the health facility, *i* is each health facility*, β* are the regression coefficients, Q is the quantity of the throughput of the health facility (doses delivered), Z represents other variables that may explain costs, and ε is the random error. Given costs are not normally distributed, the natural log of the costs and quantities was used.

### Dependent *variable*

The dependent variable in our analysis was the total annual economic cost of delivering HPV vaccines (excluding cost of vaccines and supplies) at the health facility level during the study reference period, transformed to a natural log. We also explored a regression with annual total financial costs as the dependent variable to understand drivers of financial costs and determine if these are different from drivers of economic costs.

### *Explanatory* variables

We included variables that can impact financial or opportunity costs or both (i.e., economic costs) as shown in Table 1. A key explanatory variable is the throughput of the health facility as measured by the number of doses delivered by the health facility during the reference period. Similar to the other studies, we included this variable as the natural log of the doses delivered. We also included a variable on the number of HPV vaccination sessions conducted by the health facility during the reference period. Due to positive correlation between the number of sessions conducted and doses delivered, we evaluated the impact of these variables on cost separately on most of the regressions. Other variables related to service delivery included in the regression were the number of schools served by the health facility and the median distance traveled by health facility staff to conduct school-based delivery or outreach sessions. Additional categorical variables included were whether HPV vaccine service delivery was integrated with other health services, rural vs. urban location, whether per diems were paid by the health facility for any HPV vaccination program activities conducted, and whether training and program planning activities were integrated with routine immunization or other health program activities.

**Table 1:** Sample description and explanatory variables included in the regression analysis.

**Variable name**	**Variable description**
HPV vaccine doses delivered	Number of HPV vaccine doses delivered by the health facility; included in the regression model as a natural log.
Number of HPV vaccination sessions conducted	Number of HPV vaccination sessions conducted by the health facility; included in the regression model as a natural log. *Note that this is highly correlated with the number of vaccine doses delivered, so this variable was not included at the same time as the dose delivered variable in most regressions.*
Median distance	Median distance in kilometers traveled by vaccinators to conduct HPV vaccination services to schools or communities.
Number of school-based delivery locations served	Number of school-based locations where HPV vaccine service delivery was conducted by vaccinators from the health facility.
HPV vaccine service delivery integrated	1 if HPV vaccine service delivery was reported as integrated with other service delivery such as infant vaccination, deworming, vitamin A supplementation, etc.; 0 otherwise.
Rural location	1 if the health facility is located in a rural area; 0 otherwise.
Per diems paid	1 if per diems were paid for any HPV vaccination program activity conducted by the health facility; 0 otherwise.
Number of meetings held for other activities	Total number of meetings held for program planning, social mobilization, training, and crisis management.
Program planning or training activities integrated with other programs	1 if program planning or training activities were integrated with other programs; 0 otherwise.
Vaccines collected	1 if HPV vaccines were collected from administrative-level vaccine stores; 0 if they were delivered to the health facility by the administrative-level vaccine stores.
Public transport or hired vehicles used	1 if the health facility used public transport or hired vehicles for any HPV vaccination trips to travel to vaccination sites or to collect vaccines; 0 otherwise. *Note that this variable was only included in the regression with financial costs as the dependent variable.*
Health worker utilization rate	Total person-days for health worker time spent on HPV vaccine program activities.
Non-health worker utilization rate	Total person-days for non-health worker time spent on HPV vaccine program activities.. Non-health workers include teachers and community stakeholders.
Other capital equipment on site	1 if the facility had a vehicle on site that was used by the immunization program or if they had an incinerator; 0 if they had neither a vehicle nor an incinerator.
Allocation factor for shared capital equipment	A vaccine-volume-based proportional allocation to HPV vaccines which accounts for the storage volume for HPV vaccines relative to all routine vaccines delivered at the health facility.
Program maturity	1 if country is Rwanda or Uganda, which introduced HPV vaccine in 2011 and 2015, respectively; 0 for other countries, which all introduced HPV vaccine in 2017 or later.
GDP per capita in 2019	GDP per capita for the country in 2019. This variable does not vary within the country. The variable was included as a natural log in the regression analysis and is a proxy for local price.
Salary rate for vaccinators per day	The modal salary rate used to value the time of vaccinators in the analysis. This value does not vary within the country—just across countries. This was also included as a natural log in the regression analysis.
Country dummies	1 if [country_name]; 0 otherwise (where country_name is each of the study countries).

Abbreviations: GDP, gross domestic product; HPV, human papillomavirus.

In addition, we included a count of the total number of meetings or activities held for program planning, social mobilization, training, and crisis management. Though other activities such as record keeping, waste disposal, estimating demand, and vaccine procurement were accounted for in the costing, we excluded them from this variable capturing the count of activities done. This is because these former listed activities were conducted by most health facilities in our sample, so including them would dilute the activities where more variability across health facilities appeared. We included a categorical variable on whether training and program planning activities were integrated with routine immunization or other health program activities. We limited this variable to just training and program management integration as this was explicitly asked in our study questionnaire, under the assumption that these activities could be integrated. We did not ask this question about integration for social mobilization or crisis management as these activities are typically conducted for HPV vaccination alone. We also included a variable to capture whether HPV vaccines were collected by the health facility from administrative levels or were delivered.

We included proxy variables to capture the utilization rate of health workers and non-health workers. For each type of worker, we calculated this utilization rate for each health facility as the total estimated person-time in days spent on HPV vaccine program activities during the reference period.

We also included a categorical variable capturing the availability of large capital equipment, where this variable is 1 if the health facility reported having a vehicle that is used by the immunization program or an incinerator for waste disposal. Note we excluded refrigerators for storing vaccines from this variable on capital equipment as most facilities had refrigerators, and so this variable would be 1 for most of the facilities.

Finally, we included per capita GDP (gross domestic product) and vaccinator wage rate as proxies for price of resources. Both of these proxy variables for price of resources are included as natural logs. We also included dummy variables for each country but given the correlation with the per capita GDP and wage variables, these variables were only included when the price proxy variables were excluded from the regression equation.

### Sample sizes for the analysis

Our analysis of cost drivers where the number of doses delivered was the main explanatory variable was conditional on the health facility having delivered some HPV vaccine doses during the reference period (n = 279 health facilities, including 51 of the 60 health facilities in the Ethiopia study; 40 of 43 in Guyana; all 42 in Rwanda; 55 of 56 in Senegal; all 30 in Sri Lanka; and 61 of 66 in Uganda).

### Data analysis

We provided descriptive statistics for the health facilities included in the analysis. We ran multivariate ordinary least squares regressions for each country and also on the pooled sample data. We indicated which coefficients were statistically significant at p < 0.01 and 0.01<p < 0.05 for a two-tailed test. We used the adjusted R^2^ (or the coefficient of determination) as the measure of goodness of fit of the regressions. The analysis was run using Stata version 18 (StataCorp LLC. College Station, Texas, USA). In all the multivariate regressions, we used robust standard errors by adding variance–covariance matrix of the estimators or vce(robust) specification in Stata, which corrects for any heteroscedasticity and provides a more accurate measure of the true standard error of a regression coefficient.

### Sensitivity analysis

We explored the impact of the model functional form on the results by running a regression where we included all continuous (not categorical) explanatory variables in log form. This regression is a conditional regression as any health facility for which any one of the continuous explanatory variables is zero would be dropped from the analysis given that the natural log of zero is undefined.

### Ethics review

The data used in this analysis were obtained from studies that had received ethics approvals where required: Ethiopian Public Health IRB, Rwanda National Ethics Committee, National Hospital of Sri Lanka Ethics Review Committee, Makerere University School of Public Health Research Ethics Committee (Uganda), and Uganda National Council for Science and Technology. The Guyana Ministry of Health IRB waived the protocol from IRB review. In Senegal, the study was considered program evaluation by the Ministry of Health. The study was determined to be exempt from US-based institutional review board (IRB) oversight.

## Results

Mean total economic costs per year for HPV vaccine delivery ranged from $904 for Uganda to $3,149 for Sri Lanka (Table 2) for the health facilities included in the analysis. The mean number of HPV vaccine doses delivered ranged from 170 in Guyana to 761 in Sri Lanka. In the pooled sample, each health facility provided HPV vaccination services at an average of 5 schools, but within countries, this ranged from a mean of 2 in Guyana to as many as 16 in Sri Lanka. In Ethiopia, Guyana, and Rwanda, HPV vaccine service delivery was mostly not integrated with delivery of other services, but in Senegal, Sri Lanka, and Uganda 36 %, 73 %, and 84 % of health facilities reported integration of service delivery, respectively. There were differences in urbanicity of the health facilities across countries, with 38 % of the health facilities in Guyana classified as rural compared to 70 % or above in Uganda and Rwanda. There was also a wide range in percentage of health facilities reporting whether per diems were paid for at least one HPV vaccination program activity, ranging from no per diem payment in Sri Lanka to 90 % of health facilities in Rwanda reporting per diem payments for at least one activity. Rwanda, Senegal, and Uganda had health worker utilization rates that were lower than the pooled sample mean of 52 person-days, with Sri Lanka having the highest rate (126 person-days). Not unexpected, at least 60 % of the health facilities in all countries reported that non-health workers were involved in HPV vaccination program activities. However, Ethiopia, Senegal, and Sri Lanka had non-health worker utilization rates that were larger than for the pooled sample mean of 56 person-days, with Senegal having the highest rate of 128 person-days.

**Table 2:** Characteristics of the study sample of health facilities that delivered HPV vaccine doses in 2019.

**Variable description**	**Ethiopia (n = 51)**	**Guyana (n = 40)**	**Rwanda (n = 42)**	**Senegal (n = 55)**	**Sri Lanka (n = 30)**	**Uganda (n = 61)**	**Pooled sample (n = 279)**
Mean (std. dev.) annual economic costs	$1,661 ($2,381)	$2,006 ($1,815)	$1,439 ($727)	$2,317 ($2,273)	$3,149 ($2,163)	$904 ($744)	$1,801 ($1,904)
Mean (std. dev.) HPV vaccine doses delivered	461 (4 5 1)	170 (2 3 7)	620 (3 7 2)	227 (1 8 6)	761 (7 0 1)	226 (2 1 7)	378 (4 1 8)
Mean (std. dev.) HPV vaccination sessions conducted	4 (4)	5 (4)	9 (7)	11 (9)	26 (17)	5 (6)	9 (10)
Mean (std. dev.) number of school-based delivery locations served	4 (4)	2 (2)	6 (3)	4 (3)	16 (10)	3 (3)	5 (6)
Mean (std. dev.) of the median distance	6 (6)	7 (21)	8 (6)	4 (7)	21 (16)	8 (11)	8 (13)
Percentage of health facilities reporting that HPV vaccine service delivery was integrated with delivery of other health services	6 %	13 %	5 %	36 %	73 %	84 %	37 %
Percentage of health facilities located in rural areas	49 %	38 %	71 %	51 %	N/A	74 %	51 %
Percentage of health facilities reporting that per diems were paid for HPV vaccination program activities	37 %	8 %	90 %	42 %	0 %	72 %	46 %
Mean (std. dev.) number of meetings held for other activities	8 (7)	7 (8)	6 (3)	10 (10)	26 (20)	7 (11)	9 (12)
Percentage of health facilities reporting that program planning or training activities were integrated with other programs	47 %	8 %	5 %	36 %	3 %	8 %	20 %
Percentage of health facilities reporting that they collected vaccines from administrative vaccine stores	31 %	55 %	100 %	78 %	7 %	62 %	58 %
Mean (std. dev.) health worker utilization rate	53 (42)	59 (63)	39 (34)	33 (32)	126 (1 2 3)	38 (32)	52 (61)
Percentage of health facilities reporting that non-health workers were involved in HPV vaccination program activities	78 %	83 %	67 %	89 %	80 %	62 %	76 %
Mean (std. dev.) non-health worker utilization rate	62 (1 1 0)	14 (24)	28 (34)	128 (3 5 0)	61 (73)	33 (94)	56 (1 7 4)
Percentage of health facilities reporting having other capital equipment on site	73 %	13 %	95 %	64 %	97 %	48 %	63 %
Allocation factor for shared capital equipment	0.10 (0.05)	0.48 (0)	0.08 (0.02)	0.23 (0.14)	0.25 (0.11)	0.09 (0.02)	0.19 (0.15)
GDP (std. dev.) per capita in 2019	$840 ($0)	$6,477 ($0)	$807 ($0)	$1,462 ($0)	$4,083 ($0)	$823 ($0)	$2,111 ($2,040)
Salary rate for vaccinators per day	$8 ($0)	$24 ($0)	$22 ($0)	$30 ($0)	$11 ($0)	$7 ($0)	$17 ($9)

All means are unweighted and across the whole sample (i.e., unconditional on conducting the activity and so include zero values in the means). The sample also only includes those health facilities that administered HPV vaccine doses during the reference period and reflects the sample included in the regression analysis.

Abbreviations: GDP, gross domestic product; HPV, human papillomavirus; std. dev, standard deviation.

Table 3 presents the results from the unconditional multivariate regression analysis and shows that, within countries, there is no variable (other than the health worker utilization rate) that consistently explained the variability in economic costs within and across the study countries. Higher utilization of health workers increased opportunity costs and hence increased economic costs, as shown by the positive and significant coefficients for this variable. In four of the six country regressions, non-health worker utilization rates were statistically significant, showing a positive association with economic costs. Across all countries except Rwanda and Uganda, the number of HPV vaccine doses delivered was not statistically significantly associated with economic costs in the within-country regressions. In Guyana, rural health facilities had statistically lower economic costs than urban facilities, and health facilities that collected vaccines from administrative vaccine stores had higher costs. However, in Senegal, rural facilities had statistically higher costs. In Ethiopia, health facilities that reported paying per diems for HPV vaccination program activities had statistically higher economic costs than those not paying per diems within the same country. The goodness of fit of the regressions with countries ranged from 64 % in Senegal to 90 % in Sri Lanka.

**Table 3:** Multivariate regression analysis of the economic costs against the explanatory variables.

Explanatory variable	**Ethiopia (n = 51)**	**Guyana (n = 40)**	**Rwanda (n = 42)**	**Senegal (n = 55)**	**Sri Lanka (n = 30)**	**Uganda (n = 61)**	**Pooled sample regression 1 (n = 279)**	**Pooled sample regression 2 (n = 279)**
Log of number of HPV vaccine doses delivered	0.086	0.180	0.331**	0.081	0.027	−0.152*	0.100*	0.117*
HPV vaccine service delivery integrated	−0.575	−0.018	0.055	−0.158	0.008	0.254	0.053	0.030
Number of school-based delivery locations served	−0.041	−0.039	0.039	0.013	0.008	0.052	−0.004	0.009
Median distance	0.021	−0.005	−0.0002	−0.007	0.007	0.002	0.003	0.003
Number of meetings held for other activities	0.005	0.004	0.041	0.021	0.003	0.017*	0.017**	0.019**
Program planning or training activities integrated	0.028	0.500	0.137	0.060	−0.134	−0.687	0.115	0.054
Vaccines collected	0.028	0.570*	N/A	0.015	−0.073	0.195	0.104	0.101
Rural location	0.019	−0.927**	−0.168	0.327*	N/A	−0.261	−0.166	−0.193*
Per diems paid	0.563*	0.422	0.475	0.301	N/A	0.266	0.386**	0.350**
Health worker utilization rate	0.014**	0.008**	0.006*	0.014**	0.004**	0.008**	0.007**	0.007**
Non-health worker utilization rate	0.004**	0.009**	0.006**	0.0003	0.004*	0.003	0.001	0.001
Other capital equipment on site	0.478*	1.778**	0.603	0.109	1.158	0.872**	0.530**	0.605**
Allocation factor for shared capital equipment	1.717	N/A	−2.350	1.037	1.014	5.001	1.435**	0.953**
Program maturity							−0.176	
GDP per capita in 2019							−0.067	
Salary rate for vaccinators per day							0.342**	
Ethiopia								0.310
Guyana								0.603**
Rwanda								0.848
Senegal								0.832**
Sri Lanka								Excluded comparator
Uganda								0.166
Intercept	4.672**	5.621**	3.580**	5.712**	5.282**	5.326**	4.836**	4.694**
								
R^2^	0.82	0.84	0.79	0.64	0.90	0.68	0.63	0.64

** Statistically significant at p < 0.01 for a two-tailed test.

* Statistically significant at 0.01 < p < 0.05 for a two-tailed test.

Abbreviations: GDP, gross domestic product; HPV, human papillomavirus.

For the pooled sample, some variables that were not statistically significant in the within-country regression analyses became significant. Economic costs were statistically significantly higher in health facilities where more HPV vaccine doses were delivered. A 10 % increase in doses delivered increased the economic costs by approximately 1 % in the pooled sample regressions. We also found that economic costs were approximately 40 % higher in facilities where per diems were paid compared to facilities where per diems were not paid (pooled regression 2). In addition, we found that several other factors increased economic costs and were statistically significant, specifically, when more meetings were held, when more health workers were involved in HPV vaccination program activities, where capital equipment was available at the facility, and when more shared equipment was allocated to HPV vaccine. Costs in rural facilities were statistically significantly lower than in urban facilities (pooled regression 2 but not for pooled regression 1). Per capita GDP was not statistically significantly related to economic costs (pooled regression 1) though salary rates for vaccinators were (pooled regression 1). The coefficients for service delivery integration were not statistically significant. Also, program maturity was not statistically correlated with economic costs (pooled regression 1). In pooled regression 2, which included country dummies, we found that, compared to Sri Lanka (the excluded country), economic costs were statistically significantly higher in Guyana and Senegal and the other country coefficients were not statistically significant.

We also ran the regressions with the log of the total number of sessions as the explanatory variable replacing the log of the total doses delivered in pooled regression 2 (results not shown in tables). The estimated coefficient for the number of sessions in the pooled regression was 0.203 (statistically significant at p < 0.01). This implies that a 10 % increase in the number of sessions held increases economic costs by approximately 2 %. In the regression including both the log of the total doses and the log of the number of sessions in pooled regression 2, the coefficients for number of sessions conducted remained statistically significant at p < 0.01 while the coefficient on the number of doses was not significant at p < 0.05, though significant at p < 0.10.

The pooled regression with financial costs as the dependent variable had an R^2^ of 0.43 and so was a poorer fit than the regression with economic costs as the dependent variable (not shown in the tables). The coefficient on doses delivered was not statistically significant. The only coefficients that were statistically significant were on the variables of whether per diems were paid (positive coefficient), number of school-based delivery locations served (positive), number of meetings held (positive), and whether vaccines were collected (negative). For the country dummies, only the country coefficients for Guyana and Senegal were positive and statistically significant, compared to Sri Lanka.

The conditional pooled regression, where all continuous explanatory variables were transformed to log format, included 228 health facilities for which all of the continuous variables were non-zero. This conditional regression (Supplementary Appendix 1) found that the doses delivered variable was not statistically significant but the number of school-based delivery locations served became statistically significant and was positively associated with the economic costs. Variables such as the number of meetings held, health worker and non-health worker utilization rates, availability of capital equipment at the facility, and the allocation factor for shared capital equipment remained statistically significant, as in the unconditional regression. Similar results are seen when the total number of sessions held was used as the explanatory variable instead of doses delivered.

## Discussion

Our study presents the determinants of economic costs for HPV vaccine delivery using data from six low- and middle-income countries. Similar to previous studies of routine infant vaccinations [5], [6], [8], we found that in our pooled sample unconditional regression, total doses delivered were positively and significantly associated with the economic costs, reflecting increasing resource use with an increase in service volume, in accordance with production theory. However, in the conditional regression, these variables were not statistically significant.

Our study identified cost determinants not previously explored by the studies focused on routine infant vaccinations. In our pooled unconditional regression, we found that the higher the number of HPV vaccination sessions conducted by the health facility, the higher the economic costs were for that health facility. For both the unconditional and conditional regressions, we also found that the larger the number of activities or meetings held by the health facility, the higher were its economic costs. HPV vaccination programs seeking to reduce costs may explore options to reduce the intensity of some program activities, where possible, to increase cost efficiency. In some of these countries, switching from the two-dose HPV vaccine schedule which was being implemented in 2019 to a single-dose schedule may reduce the number of HPV vaccination sessions held per health facility during the year and/or reduce the number of HPV vaccination program activities conducted (if activities are held separately for each HPV vaccine dose in the schedule). The reduction in sessions would be especially true for countries such as Rwanda where the first HPV vaccine dose is almost exclusively given in the first half of the year and the second dose is given almost exclusively in the latter half of the year [11]. Even without the schedule change, facilities can examine the activities being done and reduce intensity were possible, while holding doses delivered constant.

We found that, within and across the study countries, health worker utilization was positively and significantly associated with costs in both the unconditional and conditional regressions. Studies focused on routine infant vaccines have also found a positive association between health worker utilization and immunization program costs [5], [6]. Given that human resource time is the largest share of economic costs, reducing the intensity and frequency of activities conducted may also reduce the total human resource time spent on these activities. For the HPV vaccination program, human resource utilization also includes non-health workers, such as school staff and community stakeholders, and their engagement increases the economic costs of the program. Our primary analysis found that opportunity cost of human resource time was the largest share of economic costs [11]. In the within country regressions, we found that higher utilization of non-health workers increased economic costs in four of the six countries. However, this variable was not significant in the pooled regression. As such, in some countries, there may be a need to identify strategies to reduce the labor intensity of HPV vaccination program activities to reduce program costs.

Payment of per diems is more common for HPV vaccine delivery, as the program has characteristics of campaign vaccine delivery, even when routinized. Our primary analysis found that in countries where per diems were paid, these per diems typically were the largest share of financial costs at the health facility level [11]. In the unconditional and conditional regressions, we found that health facilities where per diems were paid for any HPV vaccination program activities had statistically higher costs than health facilities were per diems were not paid. Payment of per diems may augment health worker salaries and incentivize traveling away from health facilities to deliver services, so these expenditures may be needed to achieve program goals. However, it may reduce program costs and increase cost efficiency to not pay per diems, as is done across all health facilities in Sri Lanka.

Our study found that there were fewer statistically significant variables in the country-specific regression analyses than in the pooled sample. However, the explanatory power of these country-specific models was high. This finding may indicate that the variables we included in our model capture more of the variables that explain differences across countries than within countries. Also, there may be less variability in these variables across health facilities in the same country even though, when considered together, they influence delivery costs.

We did not find a statistically significant association between HPV vaccine service delivery integration and economic costs. It is possible that, even when integrated, costs were still borne by the HPV vaccination program. Future studies should further explore this association.

Rwanda and Uganda had the longest running HPV vaccination programs in our study, but we found no statistical association between program maturity and costs in our analysis. It is possible that the coverage improvement activities conducted in Uganda (and associated expenditures) [11] could have skewed costs upward and masked the impact of this variable. We also ran an exploratory analysis with financial costs as the explanatory variable, but this did not bring new insights, likely due to financial costs being included in economic costs and so having some of the same determinants.

Our study has several limitations. We had relatively small sample sizes, especially for the Sri Lanka sample, and this may have biased our within-country regression coefficients. There are also some factors that were included in previous analyses that we did not include here due to lack of data. Some of these variables include ownership of the facility (e.g., government, nonprofit, or private) and variables to measure the quality of services delivered by the health facility. We also did not include coverage rates as an explanatory variable due to lack of data. We used a log linear regression model for our analysis, but other regression models may have provided different findings. However, several of the few published research papers on this topic have employed a similar regression model [5], [6], [8]. Our analysis did not explore the implication of coverage improvement activities but rather costed what was done at the coverage level achieved. Therefore, we were not able to provide insights on the factors associated with coverage improvement activities. This could be a topic for future evaluations.

In conclusion, our study found several statistically significant determinants of cross-country variations in economic costs for HPV vaccine delivery. This included a positive association between costs and service volume as measured by doses delivered and sessions conducted. An increase in the intensity of conducting other program activities beyond service delivery also was positively associated with costs, as was the payment of per diems. Human resource utilization rates of health workers was strongly and positively associated with costs. Program modifications to reduce intensity and utilization rates may also reduce costs. Our findings provide evidence to HPV vaccination program stakeholders on which variables impact costs; this evidence can be used to adjust program characteristics to improve cost efficiency, especially in a context of program revitalization and coverage improvement after the pandemic.

## Source of support

This work was supported by the Bill & Melinda Gates Foundation [INV-005053]. Under the grant conditions of the Foundation, a Creative Commons Attribution 4.0 Generic License has already been assigned to the Author Accepted Manuscript version that might arise from this submission.

## CRediT authorship contribution statement

**Mercy Mvundura:** Writing – review & editing, Writing – original draft, Methodology, Formal analysis, Data curation, Conceptualization. **Rose Slavkovsky:** Writing – review & editing, Validation, Methodology, Data curation, Conceptualization. **Frédéric Debellut:** Writing – review & editing, Validation, Methodology, Data curation, Conceptualization. **Teddy Naddumba:** Writing – review & editing, Validation, Methodology, Data curation, Conceptualization. **Clint Pecenka:** Writing – review & editing, Validation, Funding acquisition, Conceptualization. **D. Scott Lamontagne:** Writing – review & editing, Validation, Methodology, Data curation, Conceptualization.

## Declaration of competing interest

The authors declare that they have no known competing financial interests or personal relationships that could have appeared to influence the work reported in this paper.

## Data Availability

The raw data used for this analysis are available on DataVerse: PATH, 2024, “HPV vaccine cost of delivery and operational context study raw data”, https://doi.org/10.7910/DVN/EVRKV3
