# Supplementary material for: Determinants of costs of human papillomavirus vaccine delivery in six low- and middle-income countries
Source: Vaccine X. 2024 Jul 27;20:100534. doi: 10.1016/j.jvacx.2024.100534 (PMC11341934; doi:10.1016/j.jvacx.2024.100534)
Supplement: Supplementary Data 1 [file mmc1.docx]

Supplementary Table 1. Multivariate regression analysis of the economic costs against the explanatory variables for the unconditional and conditional regression.

| **Explanatory variables for the unconditional regression** | **Pooled sample regression 2, unconditional (n=279)** | **Explanatory variables for the conditional regression** | **Pooled sample regression 2, conditional (n=2228)** |
| --- | --- | --- | --- |
| Log of number of HPV vaccine doses delivered | 0.117* | Log of number of HPV vaccine doses delivered | 0.016 |
| HPV vaccine service delivery integrated | 0.030 | HPV vaccine service delivery integrated | 0.003 |
| Number of school-based delivery locations served | 0.009 | Log of number of school-based delivery locations served | 0.115* |
| Median distance | 0.003 | Log of median distance | 0.026 |
| Number of meetings held for other activities | 0.019** | Log of number of meetings held for other activities | 0.108* |
| Program planning or training activities integrated | 0.054 | Program planning or training activities integrated | 0.075 |
| Vaccines collected | 0.101 | Vaccines collected | 0.077 |
| Rural location | -0.193* | Rural location | -0.038 |
| Per diems paid | 0.350** | Per diems paid | 0.275** |
| Health worker utilization rate | 0.007** | Log of health worker utilization rate | 0.446** |
| Non-health worker utilization rate | 0.001 | Log of non-health worker utilization rate | 0.190** |
| Other capital equipment on site | 0.605** | Other capital equipment on site | 0.489** |
| Allocation factor for shared capital equipment | 0.953** | Log of allocation factor for shared capital equipment | 0.207* |
| Ethiopia | 0.310 | Ethiopia | 0.072 |
| Guyana | 0.603** | Guyana | 0.793** |
| Rwanda | 0.848 | Rwanda | 0.186 |
| Senegal | 0.832** | Senegal | 0.619** |
| Sri Lanka | Excluded comparator | Sri Lanka | Sri Lanka |
| Uganda | 0.166 | Uganda | 0.010 |
| Intercept | 4.694** | Intercept | 4.174** |
|  |  |  |  |
| R^2^ | 0.64 | R^2^ | 0.79 |
